# Optimization of plasma-based BioID identifies plasminogen as a ligand of ADAMTS13

**DOI:** 10.1038/s41598-024-59672-6

**Published:** 2024-04-20

**Authors:** Hasam Madarati, Veronica DeYoung, Kanwal Singh, Taylor Sparring, Andrew C. Kwong, James C. Fredenburgh, Cherie Teney, Marlys L. Koschinsky, Michael B. Boffa, Jeffrey I. Weitz, Colin A. Kretz

**Affiliations:** 1 Department of Medicine, Thrombosis and Atherosclerosis Research Institute, McMaster University, Hamilton, ON Canada; 2 Department of Physiology and Pharmacology, Schulich School of Medicine and Dentistry, Robarts Research Institute, The University of Western Ontario, London, ON Canada; 3 Department of Biochemistry, Schulich School of Medicine and Dentistry, Robarts Research Institute, The University of Western Ontario, London, ON Canada

**Keywords:** ADAMTS13, BioID, Plasminogen, Von Willebrand factor, Cardiovascular biology, Cardiovascular models, Proteomic analysis, Blood proteins, Protein-protein interaction networks

## Abstract

ADAMTS13, a disintegrin and metalloprotease with a thrombospondin type 1 motif, member 13, regulates the length of Von Willebrand factor (VWF) multimers and their platelet-binding activity. ADAMTS13 is constitutively secreted as an active protease and is not inhibited by circulating protease inhibitors. Therefore, the mechanisms that regulate ADAMTS13 protease activity are unknown. We performed an unbiased proteomics screen to identify ligands of ADAMTS13 by optimizing the application of BioID to plasma. Plasma BioID identified 5 plasma proteins significantly labeled by the ADAMTS13-birA* fusion, including VWF and plasminogen. Glu-plasminogen, Lys-plasminogen, mini-plasminogen, and apo(a) bound ADAMTS13 with high affinity, whereas micro-plasminogen did not. None of the plasminogen variants or apo(a) bound to a C-terminal truncation variant of ADAMTS13 (MDTCS). The binding of plasminogen to ADAMTS13 was attenuated by tranexamic acid or ε-aminocaproic acid, and tranexamic acid protected ADAMTS13 from plasmin degradation. These data demonstrate that plasminogen is an important ligand of ADAMTS13 in plasma by binding to the C-terminus of ADAMTS13. Plasmin proteolytically degrades ADAMTS13 in a lysine-dependent manner, which may contribute to its regulation. Adapting BioID to identify protein-interaction networks in plasma provides a powerful new tool to study protease regulation in the cardiovascular system.

## Introduction

ADAMTS13 is a circulating plasma metalloprotease that regulates the platelet-binding capacity of Von Willebrand Factor (VWF). Insufficient ADAMTS13 activity can lead to unregulated VWF platelet-capturing and is a risk factor for thrombosis including thrombotic thrombocytopenic purpura (TTP), myocardial infarction, stroke, and venous thromboembolism^1–4^. Excessive VWF degradation by ADAMTS13 can be associated with bleeding because of insufficient VWF platelet-binding activity. Therefore, regulation of ADAMTS13 function is important to maintaining balance in the blood clotting system. VWF degradation by ADAMTS13 is regulated by shear rates, which impose force on VWF that exposes its cryptic scissile bond to ADAMTS13. These shear rates can occur during VWF secretion from endothelial cells and at sites of vessel injury, and cleavage can be enhanced when VWF binds to platelets. Recent structural studies have suggested that shear-activated VWF can overcome ADAMTS13 latency by inducing conformational changes that activate its protease activity^5^.

ADAMTS13 is unique among circulating proteases because it is constitutively secreted as an active protease and is not inhibited by natural protease inhibitors^6–8^. Despite this seemingly unregulated proteolytic activity, circulating ADAMTS13 does not prevent VWF from recruiting platelets to sites of vascular injury. This suggests that ADAMTS13 activity can be attenuated by an alternative mechanism in order to stabilize the VWF-platelet plug and initiate hemostasis. We hypothesized that plasma may contain important effector proteins that help to spatially and temporally regulate ADAMTS13 activity either through limited proteolysis or by acting as competitive ligands or inhibitors.

Proximity-dependent biotinylation is an emerging technique for discovering protein-interaction networks in biological samples. The BioID method is based on the fusion of a promiscuous biotin ligase (BirA*) to the bait protein^9^. Proteins that interact with the bait are brought into the labeling radius of the BirA* enzyme and can be covalently tagged with biotin, and subsequently identified by LC–MS/MS. Cell-based BioID studies have identified novel interactions for a host of bait proteins associated with the nuclear lamina, transcription factors, signaling pathways, and more^10–12^. However, the specificity and sensitivity of BioID assays are dependent on the expression level of the fusion protein, and the BirA* enzyme activity may be affected by local pH and ionic conditions in cells^13^. The application of BioID to cell-free biological systems may greatly expand its utility by (a) providing better control over the labeling conditions and (b) allowing labeling in non-cellular biological samples such as plasma.

In this study, the BioID method was optimized for use in plasma. Plasma BioID was then applied to identify physiological ligands of ADAMTS13 that may contribute to the regulation of its protease activity in vivo.

## Results

### Characterization of ADAMTS13-BirA* fusion protein

**Figure 1:**
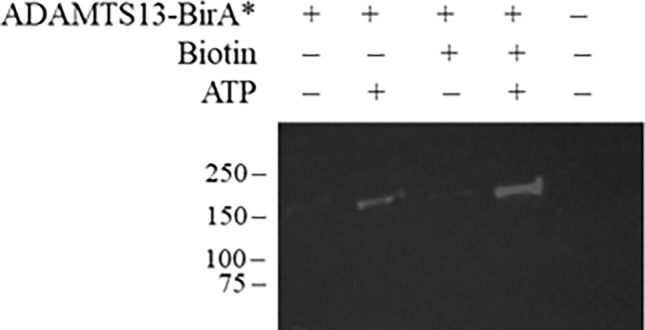
Enzymatic activity of ADAMTS13-BirA*. Self-labelling of 250 nM ADAMTS13-BirA* in HBS in the presence or absence of 50 µM biotin, 1 mM ATP. Samples were subjected to pull-down by streptavidin-agarose and visualized by Western Blot with an anti-FLAG antibody.

The C-terminus of ADAMTS13 was fused to BirA*, expressed in HEK293T cells, and purified. The specific activity of ADAMTS13-BirA* towards FRETS-VWF73 was comparable to wild-type ADAMTS13 (4.7 × 10^–3^ ± 2.3 × 10^–4^ RFU/s/nM and 4.4 × 10^–3^ ± 5.0 × 10^–6^ RFU/s/nM, respectively; *p* = 0.45), indicating that fusion to BirA* did not affect ADAMTS13 proteolytic activity. ADAMTS13-BirA* exhibited autobiotinylation activity in HEPES buffer supplemented with ATP and biotin (Fig. 1), demonstrating that the biotin-ligase activity of BirA* was also preserved when fused to ADAMTS13. Faint labelling was observed in the absence of added biotin, likely due to biotin-copurifying with the BirA* fusion protein due to their high affinity interaction, as previously described^14^. Biotin ligase activity was not observed in the absence of ATP. Therefore, the ADAMTS13-BirA* fusion protein is functional for both binding and labelling ligands of ADAMTS13.

### Optimization of plasma BioID using ADAMTS13-BirA*

Initial BioID2 assays in citrated human plasma using previously published in vitro assay conditions in buffer^15^ resulted in LC–MS/MS datasets containing low peptide counts and with poor reproducibility. Varying incubation times, temperatures, or biotin concentration did not significantly impact labeling efficiency. Furthermore, the addition of divalent cations (such as calcium, magnesium, and zinc) to plasma collected in the presence of hirudin did not significantly impact labeling efficiency (data not shown).

**Figure 2:**
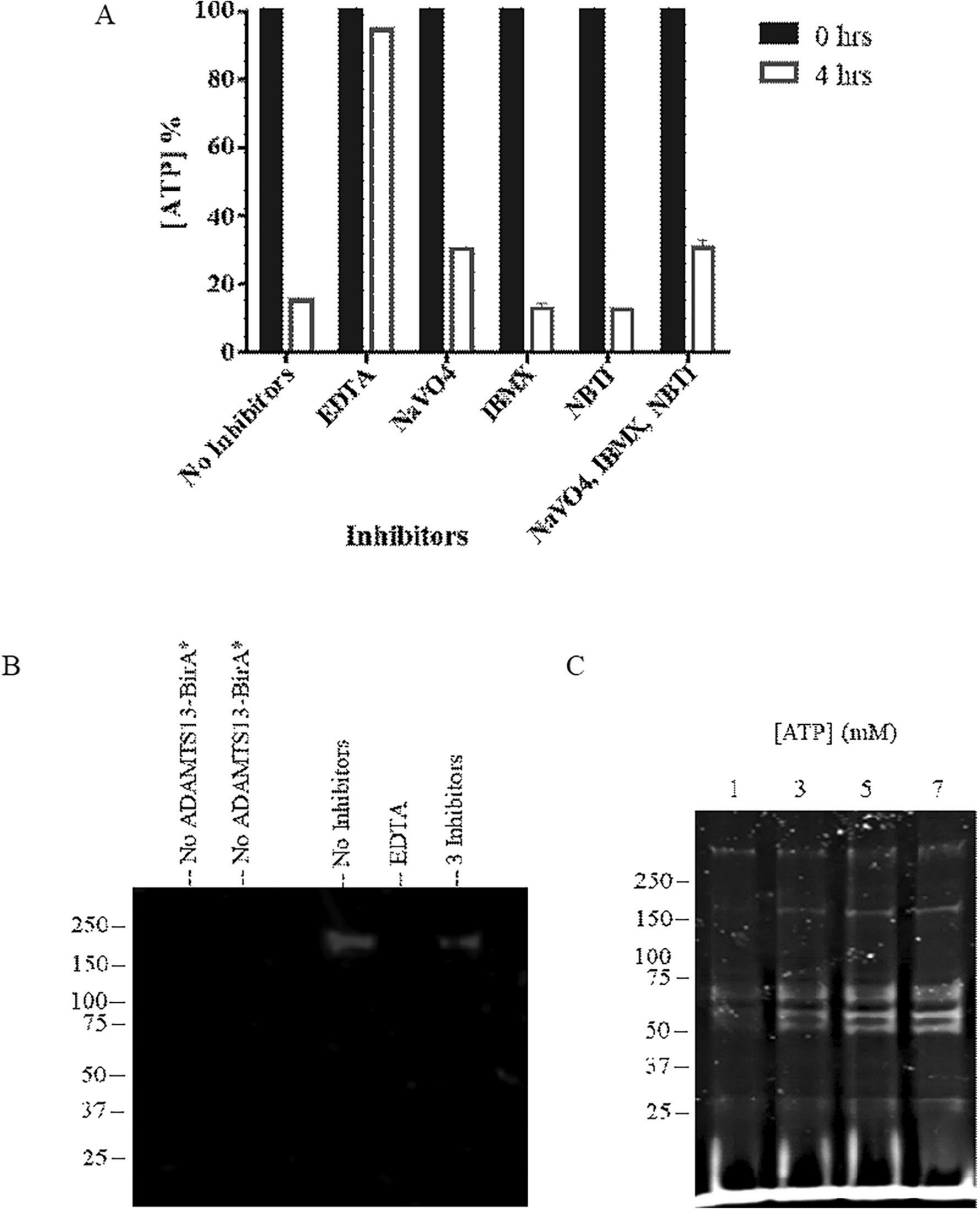
Optimization of the stability of ATP in plasma. (**A**) Measurement of ATP concentration in citrated plasma in the presence or absence of various inhibitors (3 mM EDTA, 2 mM NaVO_4_, 1 µM IBMX, 5 nM NBTI or TBS (no inhibitor)) over the course of 4 h, expressed as a percentage of that at time 0 h. (**B**) Autobiotinylation activity of 100 nM ADAMTS13-BirA* in plasma in the absence of ATPase inhibitors (TBS) or presence of ATPase inhibitors (3 mM EDTA, or 2 mM NaVO_4_, 1 µM IBMX and 5 nM NBTI) over the course of 4 h. Samples were pulled down using streptavidin agarose and visualized by Western Blot using anti-FLAG antibody. (**C**) Labelling of plasma proteins by 100 nM ADAMTS13-BirA*, with serial supplementation of 2 mM ATP every hour, after the initial addition of 1 mM ATP at the beginning of the experiment, over the course of 4 h, and visualized by SYPRO-RUBY total protein stain.

The stability of ATP in citrated human plasma was next tested using a luciferase assay to examine its impact on BioID labeling efficiency. ATP concentration rapidly declined in citrated plasma with a half-life < 5 min but was stable in a buffer for > 24 h (data not shown) consistent with previously published reports^16^, suggesting plasma ATPase activity is consuming ATP during BioID reactions. We examined the capacity of ATPase inhibitors to enhance the stability of ATP in citrated plasma. The addition of 3 mM EDTA preserved ATP for over 4 h in plasma, whereas a combination of ATPase inhibitors (Na_3_VO_4_, IBMX, and NBTI) modestly improved ATP stability (Fig. 2A). However, this cocktail of small molecule ATPase inhibitors resulted in less ADAMTS13-BirA* autobiotinylation in plasma (Fig. 2B). Assays conducted in the presence of 3 mM EDTA resulted in no detectable autobiotinylation of ADAMTS13-BirA* (Fig. 2B). These data indicate that inhibition of plasma ATPase activity can improve ATP stability in plasma but is not compatible with efficient BioID labeling reactions.

To overcome ATP instability in plasma BioID assays, ATP was serially supplemented with 2 mM additions over the 4-h reaction. The addition of ATP to plasma resulted in a dose-dependent increase in plasma protein labeling based on streptavidin pulldown and SDS-PAGE analysis (Fig. 2C). These data suggest that maintaining ATP levels throughout the labeling reaction improves biotin ligase function in plasma.

### Identification of novel plasma ligands for ADAMTS13 using BioID

**Figure 3:**
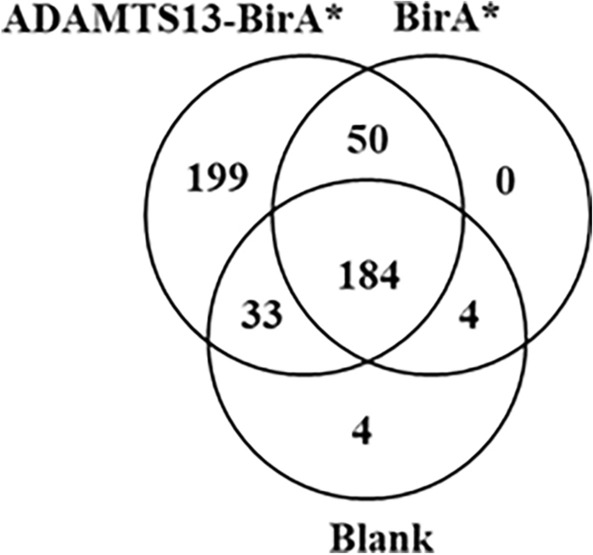
Proteins identified from the *in-vitro* BioID screen of ADAMTS13 in plasma. Venn diagram of the number of proteins labelled from BioID assay of 100 nM ADAMTS13-BirA*, BirA* or PBS buffer (no protein) in citrated plasma, with 1 mM ATP added every hour, and 50 µM biotin, at 37 °C for 4 h. Labelled proteins were isolated using agarose streptavidin beads, digested with trypsin, and identified using LC–MS/MS. The numbers represent the number of unique labelled proteins present in each condition.

Plasma BioID was performed by incubating 100 nM ADAMTS13-BirA* fusion protein or unconjugated BirA* in citrated plasma for 4 h with ATP supplementation every hour. Biotinylated proteins were extracted and identified by LC–MS/MS for 5 independent reactions^9,11^. These analyses contained an average of 225 proteins identified in the negative control (no BirA*), 238 proteins identified with unconjugated BirA*, and 466 proteins identified by ADAMTS13-BirA* (Fig. 3, Supplementary Information). There were 199 proteins uniquely identified in ADAMTS13-BirA* samples, and 50 proteins were identified in both BirA* and ADAMTS13-BirA* samples (Fig. 3). Following a one-way ANOVA, 108 proteins were significantly (*p* < 0.05) identified in the ADAMTS13-BirA* samples. Among these identified proteins, 5 are known to be extracellular (Table 1). As expected, ADAMTS13 was the most abundant protein, likely reflecting auto-biotinylation of the fusion protein. VWF was identified in the ADAMTS13-BirA* reactions, thereby serving as a positive control. The remaining extracellular proteins were selected for further investigation.

**Table 1:** Spectral counts of the extracellular proteins found to be significantly labelled by ADAMTS13-BirA*.

		ADAMTS13		BirA		No protein	
Protein	MW (kDa)	Avg	S. dev.	Avg	S. dev.	Avg	S. dev.
ADAMTS13	154	478	286.9	0	0.0	0	0.0
Vitronectin	54	78.8	28.6	31.2	5.0	27	4.1
Plasminogen	91	29.6	22.0	2.6	2.1	1.4	1.5
Von Willebrand Factor	309	1.8	1.0	0.2	0.4	0.4	0.5
Stanniocalcin-2	33	1.4	0.8	0	0.0	0	0.0

*ADAMTS* a disintegrin and metalloproteinase with thrombospondin motifs.

**Figure 4:**
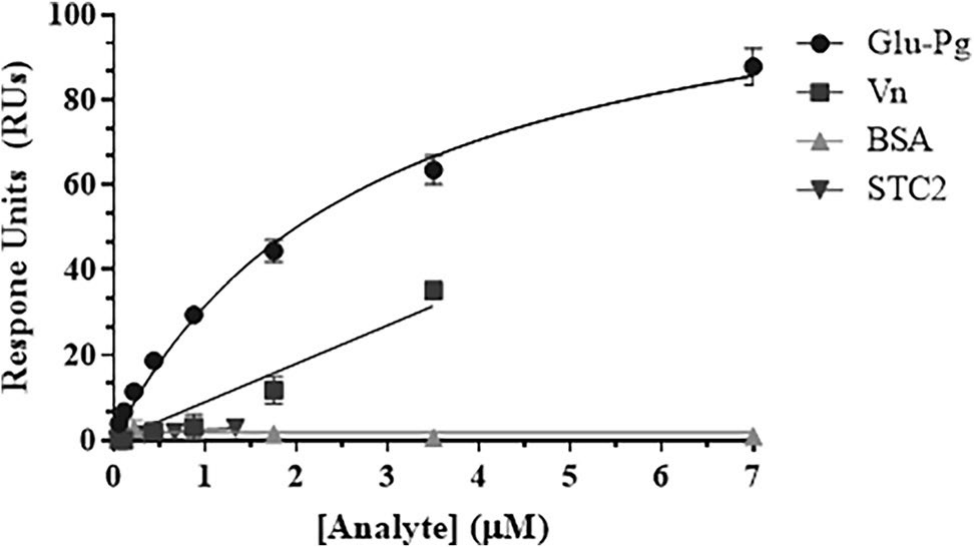
Binding of various analytes to immobilized ADAMTS13. Analytes Glu-Plasminogen (Glu-Pg), vitronectin (Vn), stanniocalcin-2 (STC2) and bovine serum albumin (BSA) were passed over immobilized ADAMTS13 at varying concentrations (0–7 µM). Change in response units was monitored over time, and corrected using a blank empty flow channel. Equilibrium value response units are plotted as a function of analyte concentration. Data points are presented as mean ± standard deviation or 3 biological replicates. Data points are fitted using a one-site binding model using GraphPad Prism.

**Figure 5:**
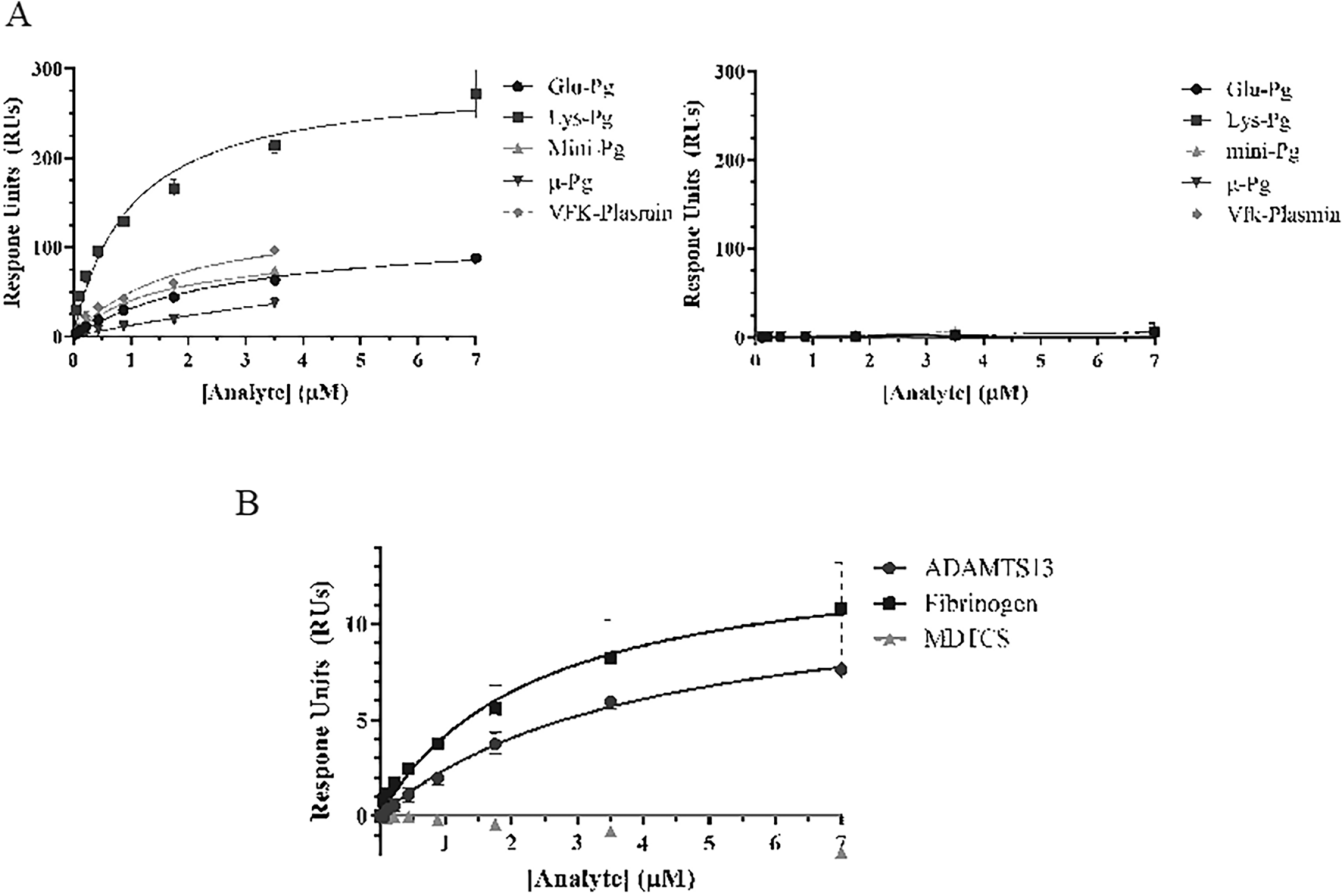
Binding of various forms of plasminogen or apo(a) to immobilized ADAMTS13, MDTCS or fibrinogen. Various forms of plasminogen (**A**) (Glu-Pg, Lys-Pg, mini-Pg, µ-Pg, VFK-plasmin) or apo(a) (**B**) at varying concentrations 0–3.5 or 0–7 µM were injected onto immobilized ADAMTS13 (**A**—left), MDTCS (**A**—right), or fibrinogen. Change in response units was monitored over time, and corrected using a blank empty flow channel. Equilibrium value response units are plotted as a function of analyte concentration. Data points are presented as mean ± standard deviation or 3 biological replicates. Data points are fitted using a one-site binding model using GraphPad Prism.

Vitronectin was identified in both ADAMTS13-BirA* and BirA* BioID assays, whereas stanniocalcin-2 was detected only by ADAMTS13-BirA* (Table 1). SPR-based binding assays with vitronectin demonstrated a modest increase in response units to immobilized ADAMTS13 that did not achieve saturation up to 3.5 µM, suggesting a weak or non-specific interaction (Fig. 4). Stanniocalcin-2 did not exhibit any binding to immobilized ADAMTS13 up to 1.3 µM (Fig. 4). Glu-plasminogen exhibited dose-dependent, saturable, and reversible binding to immobilized ADAMTS13 (Fig. 5).

### Mapping the interaction between ADAMTS13 and plasminogen

Plasminogen is a zymogen composed of 5 kringle domains (KI, KII, KIII, KIV, and KV) and a protease domain (P) and can adopt a closed (Glu-plasminogen) or an open (Lys-plasminogen) form^17^. Lys-plasminogen bound to immobilized ADAMTS13 more tightly than Glu-plasminogen, with K_D_ values of 1.0 ± 0.2 and 2.8 ± 0.3 µM respectively (Fig. 5A). VFK-plasmin, the activated form of plasminogen with a blocked active site, bound to immobilized ADAMTS13 with a K_D_ value of 1.8 ± 0.6 µM (Fig. 5A). Truncated forms of plasminogen were next tested for binding to ADAMTS13 to partially map the binding interaction. Mini-plasminogen (containing KV-P) bound to immobilized ADAMTS13 with a K_D_ value of 1.5 ± 0.4 µM, whereas micro-plasminogen (containing P) exhibited weak and non-saturable binding (Fig. 5A). No forms of plasminogen bound to immobilized MDTCS, a C-terminal truncation variant of ADAMTS13 lacking 7 Tsp-1 repeats and 2 CUB domains (Fig. 5A). These data suggest that ADAMTS13 preferentially binds to Glu- and Lys-plasminogen and implicate the plasminogen KV domain and the C-terminal Tsp-1 repeats or CUB domains of ADAMTS13 as the likely points of interaction.

Other kringle domain-containing plasma proteins, such as prothrombin, FXIIa, or tPA did not bind to immobilized ADAMTS13 (data not shown), suggesting that the interaction with plasminogen is specific and not a general feature of kringle domains. Apolipoprotein(a) (apo(a)) exhibits close sequence homology to plasminogen and is comprised of a variable number of kringle IV-like domains, followed by kringle V and a non-functional protease domain^18,19^. Apo(a) bound to immobilized ADAMTS13 in a dose dependent and saturable manner with a K_D_ value of 4.0 ± 0.6 µM. By comparison, apo(a) bound fibrinogen with a K_D_ value of 2.4 ± 0.6 µM (Fig. 5B). Like plasminogen, apo(a) did not bind immobilized MDTCS (Fig. 5B). These data demonstrate that common kringle domain features between apo(a) and plasminogen mediate their interactions with full length ADAMTS13.

**Figure 6:**
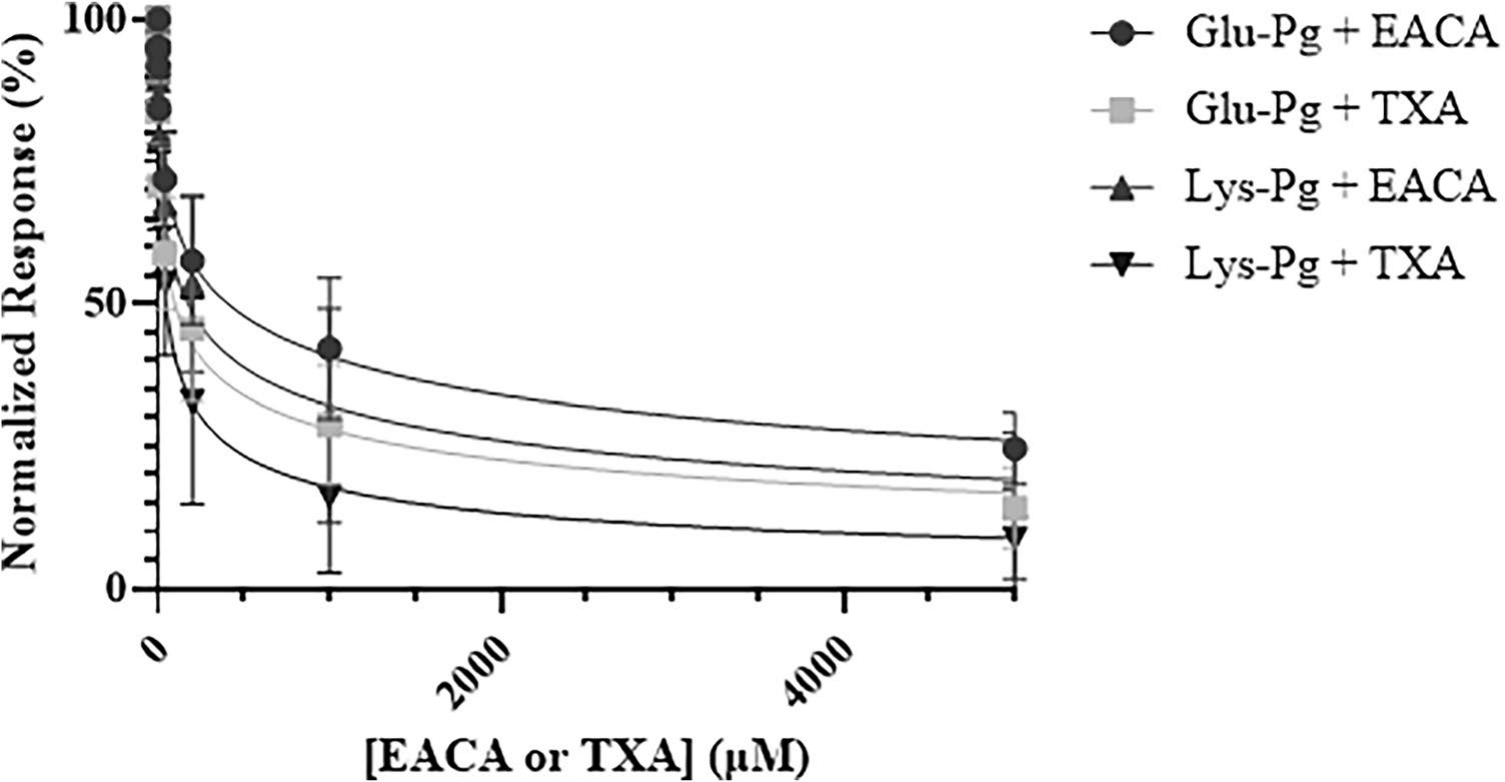
Effect of EACA or TXA onto Glu-Pg and Lys-Pg binding to immobilized ADAMTS13. Increasing concentration (0–5 mM) of EACA or TXA were injected along with 2 µM Glu-Pg or Lys-Pg onto immobilized ADAMTS13. Change in response units was monitored over time, and corrected using a blank empty flow channel. Equilibrium value response units are plotted as a function of analyte concentration. Data points are presented as mean ± standard deviation or 3 biological replicates. Data points are fitted using a one-site binding model using a competitive inhibition model ([inhibitor] vs normalized response—variable slope) using GraphPad Prism (V9).

The kringle I, kringle IV, and kringle V domains of plasminogen are known to bind to lysine residues on target proteins, like fibrin(ogen)^20^. Lysine analogs such as ε-aminocaproic acid (EACA) or tranexamic acid (TXA) compete with fibrin(ogen) for binding to plasminogen^21^. Both EACA and TXA dose-dependently attenuated plasminogen binding to immobilized ADAMTS13 (Fig. 6). TXA attenuated Glu-plasminogen or Lys-plasminogen binding to immobilized ADAMTS13 with IC_50_ values of 98.1 ± 23.0 and 46.7 ± 12.2 µM, respectively. EACA was less potent, displacing Glu-plasminogen or Lys-plasminogen from immobilized ADAMTS13 with IC_50_ values of 409.6 ± 96.5 and 175.2 ± 69.0 µM, respectively. Furthermore, TXA dose-dependently attenuated apo(a) binding to immobilized ADAMTS13 and fibrinogen, with IC_50_ values of 96.0 ± 61.7 and 92.0 ± 67.3 µM, respectively. These data indicate that the C-terminal domain of ADAMTS13 binds to the kringle domains of plasminogen and apo(a) in a lysine-dependent manner.

**Figure 7:**
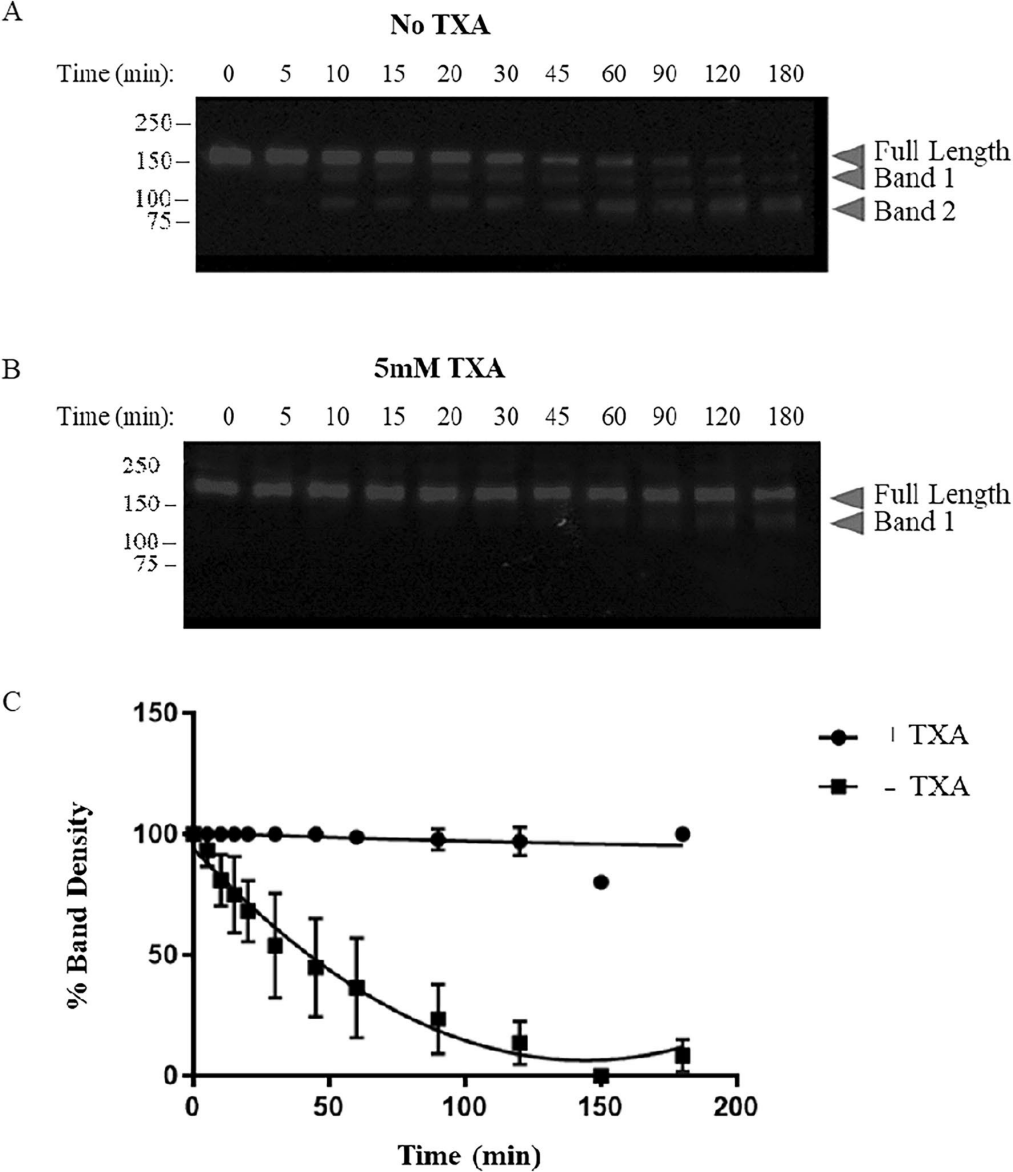
Western blot analysis of ADAMTS13 degradation by plasmin, with or without TXA. 100 nM ADAMTS13 was incubated with 10 nM plasmin in the absence (**A**) or presence (**B**) of 5 mM TXA. Aliquots were removed at various time points into reducing sample buffer and boiled immediately to stop the reaction. Samples were analyzed by Western Blot using an antibody targeting the metalloprotease domain of ADAMTS13. Bands at approximately 180 kDa correspond to full-length ADAMTS13, and the appearance of other bands indicates degradation of ADAMTS13 into smaller fragments. (**C**) The relative intensity of the intact ADAMTS13 band was determined at each time point and plotted as a function of time. Data were fit to a one phase exponential decay model using GraphPad Prism (V9). Values represent the mean ± standard deviation of 3 biological replicates.

Plasmin, the active form of plasminogen, is known to cleave ADAMTS13 and reduce its capacity to regulate VWF multimer length^22^. To evaluate the contribution of kringle domains to plasmin degradation, ADAMTS13 was incubated with plasmin in the absence or presence of 5 mM TXA. As expected, plasmin degraded ADAMTS13 in a time-dependent manner, resulting in fragmentation patterns consistent with cleavage sites within the Tsp-1 repeats (Fig. 7). The presence of TXA protected ADAMTS13 from plasmin degradation (Fig. 7). Plasmin degraded ADAMTS13 at a rate of 1.6 ± 0.1 nM/min, which was reduced to 0.05 ± 0.02 nM/min in the presence of TXA. Therefore, TXA attenuated plasmin-mediated ADAMTS13 degradation by 34-fold, suggesting that the capacity for plasmin to degrade ADAMTS13 is dependent on lysine-binding kringle domains.

## Discussion

We optimized BioID2 to identify novel plasma ligands of the metalloprotease ADAMTS13. Proximity-dependent biotinylation techniques have been used to identify protein interaction networks in cells and in complex organisms^12,23^. However, the stability of ATP is a bottleneck in plasma-based BioID2 applications because of the high ATPase activity in whole blood and plasma^16^. ADAMTS13-BirA* autobiotinylation was detected in plasma with 1 mM ATP, however this condition did not result in sufficient peptide identification in the mass spectrometry data to identify potential ligands. Although ATPase inhibitors prolonged the half-life of ATP in plasma, they also inhibited the enzymatic activity of BirA*, consistent with the requirement for ATP hydrolysis in the conversion of d-biotin into the 5’-biotinyl-AMP intermediate that covalently binds to target ligands^24^. Serial supplementation of ATP is essential to maintain its availability to the BioID2 BirA* enzyme in plasma and improve the yield of mass spectrometry data.

The plasma ADAMTS13-BioID2 screen revealed 108 significantly detected proteins compared to unconjugated BirA* alone. ADAMTS13-BirA* samples were enriched for cell-associated proteins like actin, tubulin, and other metabolic and cell cycle proteins, which may reflect labeling of receptors on the surface of plasma microparticles^25^. Previous studies have demonstrated ADAMTS13 binding to endothelial cells^26^ and platelets via the CD36 scavenger receptor^25^, which may serve to enhance its capacity to degrade VWF-platelet strings following vascular injury. ADAMTS13-BirA* labelling of CD36 on the surface of plasma microparticles released from these or other cells may have enriched microparticle-associated proteins including intracellular and membrane-associated proteins. Data collected from the unconjugated BirA* control showed virtually no intracellular protein labelling, consistent with this proposed mechanism. The consequences of ADAMTS13 binding to CD36 is not well explored in the literature and may be worth revisiting in light of this potential explanation of our observation. Additional experiments were considered to test this interpretation of the data, but ultimately not pursued because of artifacts that would be introduced into the system. For example, performing the BioID reaction in plasma containing membrane-disrupting detergents may lead to changes in protein-folding in either plasma proteins or the ADAMTS13-BirA* fusion protein that do not represent their native conformation. Alternatively, ultracentrifugation of the plasma samples to removed microparticles, may also remove microparticle associated plasma proteins and lipoproteins that could obscure our capacity to detect novel plasma ligands of ADAMTS13. VWF was expected to be among the most abundant extracellular proteins labelled by ADAMTS13-BirA* because of its known association with ADAMTS13 and its high plasma concentrations. The relatively weak VWF labelling may reflect the static conditions under which the labelling reaction was performed. VWF and ADAMTS13 are known to engage in a shear-dependent binding mechanism^27,28^. Only ~ 3% of ADAMTS13 is bound to VWF in platelet-poor plasma in the absence of shear forces^27,28^. Whether the fusion of BirA* to the CUB domains of ADAMTS13 also attenuated the interaction with VWF, or introduced a conformation in ADAMTS13 that led to off-targeted labelling remains unknown. Future studies may examine ADAMTS13-BirA* plasma labelling in a high shear-environment^29^.

Plasminogen was the most abundant plasma protein labeled by ADAMTS13-BirA*, suggesting that it is an important ligand of ADAMTS13 in plasma. This observation supports a previous yeast two-hybrid screen that identified Lys-plasminogen as a ligand for ADAMTS13^30^. Similar to this study, we observed full length ADAMTS13 bound Lys-plasminogen with higher affinity than Glu-plasminogen, indicating preferential binding to the kringle domains that are obstructed by the closed conformation adopted by Glu-plasminogen^31^. Furthermore, ADAMTS13 bound mini-plasminogen and not micro-plasminogen, suggesting the high affinity lysine-binding KV as a potential binding site. We observed no binding of MDTCS to plasminogen analogues, suggesting the binding site resides in its C-terminus, and not the Cys-rich or spacer domains as previously suggested^30,32^. ADAMTS13 does not have a C-terminal lysine residue, which is responsible for the high affinity binding of plasminogen to ligands such as fibrin. Therefore, plasminogen likely binds to a lysine residue on an exposed surface loop on ADAMTS13. Previous studies have suggested that plasmin may be an important regulator of ADAMTS13^22,33,34^. Crawley et al. demonstrated that plasmin proteolytically removes the C-terminal CUB domains from the proximal catalytic domains of ADAMTS13. While removal of C-terminal CUB domains can increase the activity of ADAMTS13 toward small biochemical substrates like VWF73^35^. It reduces the capacity of ADAMTS13 to cleave VWF-platelet strings in vivo^22,34,36^. This activity may be relevant in human disease because plasmin activity has been observed in patients during acute episodes of thrombotic thrombocytopenic purpura (TTP)^33^. In addition, ADAMTS13 has been found with similar degradation patterns in plasma collected from patients with DIC associated with sepsis^37^, suggesting that proteolytic degradation may serve to regulate ADAMTS13 activity in vivo. Whether ADAMTS13 degradation in these conditions is due to plasmin activity remains to be determined. Binding to lysine-dependent kringle domains was confirmed using ε-aminocaproic acid (EACA) and tranexamic acid (TXA) which blocked binding of ADAMTS13 to plasminogen and protected ADAMTS13 from degradation by plasmin. TXA is used clinically to treat patients with severe bleeding by attenuating plasminogen activation and fibrin clot dissolution, and its use has not been associated with increased risk of thrombosis^38^. Our data suggest that TXA may also serve to protect ADAMTS13 from plasmin degradation. Future studies should examine whether TXA co-administration can protect recombinant ADAMTS13 from plasmin degradation during acute episodes of TTP in experimental animal models. Increasing the therapeutic efficacy of recombinant ADAMTS13 infusion in TTP patients using TXA may help to prevent relapse and reduce the number of plasma exchange treatments required to achieve remission. In addition, engineering mutants of ADAMTS13 that are protected from proteolytic degradation may improve thrombolysis therapy when administered in combination with tPa. Future studies will examine these concepts in experimental thrombosis models.

Apo(a) is found in association with LDL-like particles in circulation to form lipoprotein(a), and its levels are associated with the risk of myocardial infarctions^39,40^. Previous studies have demonstrated that low levels of ADAMTS13 are associated with an increased risk of myocardial infarction through the influence of lipid levels^41^. Specifically, ADAMTS13 levels were positively correlated with total cholesterol and triglycerides, and negatively correlated with high-density lipoprotein-cholesterol levels^41^; lipoprotein(a) levels were not determined in this study. Lp(a) levels in the general population can range substantially from 5 nM to greater than 500 nM^42^. However, approximately 80% of the population have Lp(a) levels of less than 100 nM^42^. Apo(a) was not identified as a ligand of ADAMTS13 in the BioID assay. It is possible that this was due to the low levels Lp(a) in the pooled plasma samples. In comparison to plasminogen, apo(a) contains a strong lysine binding site in KIV type 10, which shares the highest homology to plasminogen’s KIV domain, and weak lysine binding sites in KIV types 5–8 and KV^43^. The KIV type 10 site is partially occluded in apo(a) by an intramolecular interaction analogous to—but likely structurally distinct from—the closed conformation of Glu-plasminogen^44^. KIV types 5–8 lysine binding sites are blocked in apo(a) in the context of Lp(a) due to non-covalent interactions with apoB-100 in the lipoprotein particle. ADAMTS13 bound apo(a) with a lower affinity than Glu-plasminogen, indicating preferential binding to plasminogen. Furthermore, apo(a) binding to ADAMTS13 was attenuated by TXA with a similar IC50 as Glu-plasminogen, which may indicate the preferential binding of ADAMTS13 to plasminogen involves more than the lysine-binding site. Hence, in competition between plasminogen and apo(a), plasminogen will likely preferentially bind to ADAMTS13. Future studies will examine the capacity of ADAMTS13 to associate with lipoprotein(a), and the influence of this interaction on the capacity of ADAMTS13 to cleave VWF.

Overall, this study advances the use of proximity-dependent biotinylation techniques, like BioID, to identify protein interaction networks in plasma and other acellular environments. BioID technology is rapidly evolving with new and more efficient biotin ligases that may increase the sensitivity of the technique in the future. Recently. Banon et al. performed directed evolution of the BirA* enzyme from *E.coli* to generate variants with improved labelling efficiency^45^. These variants, termed TurboID and MiniTurboID, reduce the labelling time of BirA* from 18 h to 10 min with greater specificity and sensitivity in target identification. This drastic reduction in labelling time may be particularly beneficial in plasma-BioID experiments where rapid consumption of ATP in plasma can reduce labelling efficiency. Therefore, future studies should test whether TurboID or MiniTurboID are more effective at identifying novel ligands of plasma proteins like ADAMTS13. Rapid labelling times may also facilitate the discovery of novel protease substrates that are expected to exhibit more transient binding kinetics due to substrate turnover. The successful application of plasma-BioID to ADAMTS13 represents a new advancement in BioID by enabling the exploration of protein-interaction networks in cell-free environments that may uncover new pathways of regulation in the cardiovascular system.

## Experimental procedures

### Vector design of ADAMTS13-BirA* and BirA*

Vector and primer design of the fusion ADAMTS13-BirA* and unconjugated BirA* were performed using SeqBuilder and IDT analyzer. Fragments of FLAG-tag and Kozak sequence were added to the assembly during the extraction process for identification and better expression of both proteins. DNA fragments of the full-length constructs were extracted using Phusion High-Fidelity polymerase (NEB: M0530S) under the following conditions: < 250 ng template DNA, 1 µM primer 1, 1 µM primer 2 (Supplementary Information—Table 2), Phusion High-Fidelity MasterMix and _dd_H_2_O. The thermal cycles consisted of: (1) 95 °C, 3 min; (2) 95 °C, 30 s; (3) 62 °C, 30 s; (4) 72 °C, 30 s/kb; (5) 72 °C, 10 min; repeating steps 2–4 for a total of 30 cycles. The reactions were assessed on 1% agarose gel, and the fragments were purified using a Qiagen gel extraction kit (Qiagen: 28704) or DNA clean-up kit (Qiagen: 28104). The fragments were quantified using NanoDrop and assembled into pcDNA 5.1 FRT containing a C-terminal his tag using Gibson assembly (NEB: E5510S). Correct assembly was determined using PCR at every junction site (Supplementary Information—Table 3) and sequenced using Sanger sequencing at MobixLab (McMaster University).

### Expression of ADAMTS-BirA* and BirA*

The cloned vectors were transfected into HEK 293TRex using the Flp-In TRex system (ThermoFisher Scientific: K650001) and Lipofectamine 3000 (Invitrogen: L3000-15) and selected against Hygromycin B to establish a constitutively expressing stable cell line. Healthy cells were propagated into roller bottles using complete media (DMEM + 10% FBS + Pen/Strep) and the proteins were expressed in Freestyle media. The media was collected, filtered, purified using Q Sepharose (Sigma Aldrich: GE17072901) at 1 M NaCl in Tris buffer (pH 8.0), and HisPur Ni–NTA Resin (Thermo Scientific: 88221) at 250 mM imidazole in 0.5 M NaCl and Tris buffer (pH 8.0), buffer exchanged into PBS buffer (137 mM NaCl, 2.7 mM KCl, 10 mM Na_2_HPO_4_, and 1.8 mM KH_2_PO_4_) using PD-10 desalting columns (GE Healthcare: 17-0851-01), then analyzed for protein expression using gel electrophoresis (SDS-PAGE), total protein stain (SYPRO-RUBY) and western blots (anti-Flag-HRP; Sigma: SLBP6984V).

### Measurement of ADAMTS13 activity

ADAMTS13 activity was quantified using a standardized fluorescent-labeled substrate, FRETS-VWF73 (AnaSpec: AS-63728-01). ADAMTS13 (2.5 nM) as wild-type (R&D Systems: 6156-AD) or as ADAMTS13-BirA* was reacted with FRETS-VWF73 (1 µM) in FRETS buffer (20 mM Tris–HCl, 25 mM CaCl_2_, 0.05% Tween-20, pH 7.4) and the activity was measured using SpectraMax M3 (Molecular Devices) fluorescence mode at ex = 340 nm and em = 450 nm. The proteolysis rate was calculated by linear regression of the initial slope of the measured activity using SoftMax Pro (v5.4). ADAMTS13 specific activity rate was calculated by linear regression of the slope of the change in proteolysis rate over the change in concentration of ADAMTS13 and plotted using GraphPad Prism (v. 6). The concentration of ADAMTS13 utilized ranged from 0.5 to 10 nM, with a replicate number of 2.

### In-vitro BioID screen of ADAMTS13 in plasma

Total reaction volumes of 100 µL containing ADAMTS13-BirA* or BirA* (100 nM) or no protein (PBS—phosphate-buffered saline: 137 mM NaCl, 2.7 mM KCl, 10 mM Na_2_HPO_4_, and 1.8 mM KH_2_PO_4_), containing 90 µL citrated pooled normal plasma (PNP), ATP (1 mM / hour) and biotin (50 µM), were made and incubated for 4 h on a top-over-bottom rotator at 37 °C. Note that ATP supplementation could be done more frequently than 1 h, as the timing of this was not extensively studied. 10–20 µL of streptavidin-agarose bed volume (Thermo Scientific: 20349) was washed 3 × in 10 × bed volume BioID wash buffer (TBS (Tris-buffered saline: 20 mM Tris–HCl and 150 mM NaCl) + NaCl: 20 mM Tris–HCl, 450 mM NaCl, pH to 7.6), then pelleted at 500 xg for 3 min, and was added to the plasma mixture along with BioID wash buffer up to 1 mL. Pull-down was performed on a top-over-bottom rotator at room temperature for 3 h. The Pull-down resin was then washed 5 × in 1 mL BioID wash buffer for 3 min in a top-over bottom rotator at room temperature and pelleting the resin at 500 xg for 3 min. 25 µL of SDS-PAGE reducing loading dye (β-mercaptoethanol included) was added to the beads and the mixture was heated at 95 °C for 5 min. The samples were then loaded onto a 4–20% polyacrylamide gel (Bio-Rad: 456–1096) and were separated using SDS-PAGE followed by analysis through total protein stain and/or western blot. Previous studies have shown that long-term exposure of ADAMTS13 to sodium citrate leads to a decrease in its activity^46^. Attempts to use other anticoagulants, like heparin, were not successful because of protein-binding artifacts.

### LC/MS/MS analysis of enriched labeled proteins

For mass spectrometry (LC/MS/MS) analysis, the *in-vitro* BioID screen was performed as previously described with the exception in the use of 1 mL of plasma and the plasma mixture was mixed up to 2 mL with BioID wash buffer for the wash steps. Three additional washes of 50 mM NH_5_CO_3_ (pH 8.0) were made before the samples were processed by SPARC-BioCentre (Sick Kids Hospital, Toronto) by urea/DTT treatment, alkylated using iodoacetamide, trypsin-digested, separated, and analyzed using liquid chromatography-tandem MS (LC/MS/MS) as described^9,11^. Results were analyzed using Scaffold 4 (Proteome Software). Significance was calculated between ADAMTS13-BirA* and BirA* samples, and between ADAMTS13-BirA* and Blank samples, using a one-way ANOVA with posthoc Tukey HSD test through RStudio. The p-value of less than 0.05 in both comparisons was deemed significant.

### Measuring ATP concentration

ATP in plasma was measured using the CellTiter-Glo 2.0 assay (Promega: G9241) and analyzed using SoftMax Pro (v.5.4) and GraphPad Prism 6. The 100-µL reactions consisted of: 100 nM of biotin ligase (ADAMTS13-BirA*, BirA*, or N/A), 90 µL citrated PNP pre-warmed to 37 °C, 1 mM ATP, 50 µM biotin, and various ATPase inhibitors: 3 mM EDTA, 2 mM Na_3_VO_4_ (pH 10), 1 µM IBMX, 5 nM NBTI, or TBS (no inhibitor). Reaction time began at the addition of ATP and at each time point, 5 µL of the reaction was diluted in 370 µL of TBS and placed in liquid nitrogen for a quick freeze. The reaction was stopped at 4 h or as designated. Frozen aliquots were thawed on a benchtop and mixed at a 1:1 ratio of sample to Luciferase (40 µL Cell Titre Glo2 reagent, 3 mM MgCl_2_). Samples were read in a 96-well opaque plate as previously described alongside a standard curve of ATP (0.1–100 µM) prepared in TBS. The initial and final concentrations of ATP were normalized to a percentage of the initial ATP concentration and was plotted using GraphPad Prism (v. 6).

### Binding studies using surface plasmon resonance (SPR)

The affinities of soluble full-length recombinant ADAMTS13 and truncated ADAMTS13 (MDTCS) (R&D Systems: 6156-AD-020 and 4245-AD-020 respectively), and various purified proteins including plasminogen (Pg: Glu-, Lys-, Mini-, µ-), inactivated plasmin (VFK-Plasmin), vitronectin (Vn), and stanniocalcin-2 (STC2) were determined by surface plasmon resonance (SPR) on a Biacore T200 (Cytiva). Biacore sensor chips (CM5), an amine coupling kit, and acetate buffer (pH 5.0) were from Cytiva. Commercially supplied vitronectin (Molecular Innovations: HVN-U) was utilized. Stanniocalcin-2 was expressed in HEK 293T cells and purified using Q Sepharose and Ni–NTA IMAC. Plasminogen and its derivatives were prepared as previously described^47^. A 17-kringle form of apo(a) was expressed in HEK 293 cells and purified from conditioned medium harvested from these cells by lysine-Sepharose affinity chromatography, as previously described^48^. Full-length ADAMTS13 and MDTCS were immobilized onto separate flow cells of a CM5 sensor chip at pH 4.0 using amine-coupling to 500–1000 response units (RU). Flow cell 1, blocked with ethanolamine, was used as the reference.

Binding experiments were performed in 20 mM HEPES–NaOH, 150 mM NaCl, pH 7.4 (HBS) containing 0.01% Tween-20, 10 mM CaCl_2_, and 10 µM ZnCl_2_ at a flow rate of 20 µl/min at 25 °C. Increasing concentrations of analyte, 0–7 µM (unless otherwise specified), containing 5 mM AEBSF (Sigma-Aldrich: 30827-99-7) and 10 µM VFKck (Sigma-Aldrich: 627624), were injected for 60 s (association), followed by running buffer alone for 300 s (dissociation). Flow cells were regenerated with 0.5 M NaCl for 60 s. Sensorgrams were analyzed with BIAcore T200 evaluation software (v 1.0) and equilibrium binding values were transferred to GraphPad Prism (v. 6), whereby dissociation constants were determined from the non-linear regression fit of a one-site binding model. This model uses the equation *y* = *Bmax* ∗ (*x*/*KD* + *x*), where y is the total binding, x is the concentration of ligand, B_max_ is the maximum specific binding, K_D_ is the equilibrium dissociation constant which is the concentration of ligand at half-maximum binding at equilibrium.

Competition binding experiments were performed under the same conditions. The analytes, 2 µM Lys-Pg, Glu-Pg, or apo(a), containing 5 mM AEBSF, 10 µM VFKck, and varying concentrations of 0–5 mM ε-aminocaproic acid (EACA) (Sigma-Aldrich: 60-32-2) or tranexamic acid (TXA) (Sigma-Aldrich: 1197-18-8) were injected. IC50 (concentration of inhibitor that gives 50% binding of analyte) constants were determined from non-linear regression fit of a “[inhibitor] vs normalized response—variable slope” model. This model uses the equation *y* = *Bottom* + (*Top* − *Bottom*)/(1 + (*IC*50/*X*)^*SlopeFactor*^), where y is the normalized total binding (to 100%), x is the concentration of inhibitor, IC50 is the concentration of inhibitor at half binding of analyte at half-maximum binding at equilibrium, SlopeFactor is the steepness of the curve (-1 is standard), and Top and Bottom are plateaus in the units of Y axis, where Bottom is forced to value of 0.

### ADAMTS13 cleavage assay

*In-vitro* proteolysis reactions of ADAMTS13 occurred at a volume of 100 µL using full length-ADAMTS13 (R&D Systems: 6156-AD-020) in ADAMTS13 reaction buffer (20 mM Tris–HCl, 150 mM NaCl, 10 mM CaCl_2_, 10 μM ZnCl_2_, 0.05% Tween-20, pH 7.4). ADAMTS13, at 100 nM, was incubated for varying lengths of time (0–3 h) with 10 nM plasmin (#HCPM-0140, Prolytix, Essex Junction, VT), at 37 °C. Reactions occurred without TXA, or after pre-incubation of plasmin with 5mM TXA for 5 min. Reactions were stopped using SDS-loading dye and separated via 4–20% polyacrylamide SDS-PAGE under reducing conditions. SDS-PAGE gels were analyzed through a western blot using polyclonal anti-ADAMTS13 antibody (Abcam: ab28274) and goat anti-rabbit HRP-conjugated antibody (Bir-Rad: 1706515). Imaging was performed on the ChemiDoc Imager System (Bio-Rad), and densitometric analysis of western blots was performed using the Image Lab Software (Bio-Rad, version 5.2.1).

### Supplementary Information

Supplementary Information 1.

Supplementary Information 2.

Supplementary Information 3.

Supplementary Information 4.

Supplementary Information 5.

Supplementary Information 6.

## Data Availability

The mass spectrometry proteomics data have been attached as Supplementary Materials as a Scaffold file and excel file.

## Abbreviations

ADAMTS13: A disintegrin and metalloprotease with a thrombspondin type 1 motif member 13
VWF: Von Willebrand factor
SPR: Surface plasmon resonance
PPI: Protein–protein interactions
BioID: Proximity-dependent biotinylation
ATP: Adenosine triphosphate
AMP: Adenosine monophosphate
bio-AMP: Biotinoyl-intermediate
ECM: Extracellular matrix
Na_3_VO_4_: Sodium orthovanadate
IBMX: 3-Isobutyl-1-methylxanthine
NBTI: S-(4-nitrobenzyl)-6-thioinosine
EDTA: Ethylenediaminetetraacetic acid
PBS: Phosphate-buffered saline
TBS: Tris-buffered saline
RU: Response units
Pg: Plasminogen
BSA: Bovine serum albumin
FXII: Factor 12
EACA: Epsilon-aminocaproic acid
TXA: Tranexamic acid
LC/MS/MS: Liquid chromatography with tandem mass spectrometry
HEK: Human embryonic kidney
FBS: Fetal bovine serum
DTT: Dithiothreitol
