# Supplementary figures and images for: Optimization of plasma-based BioID identifies plasminogen as a ligand of ADAMTS13
Source: Sci Rep. 2024 Apr 20;14:9073. doi: 10.1038/s41598-024-59672-6 (PMC11032339; doi:10.1038/s41598-024-59672-6)

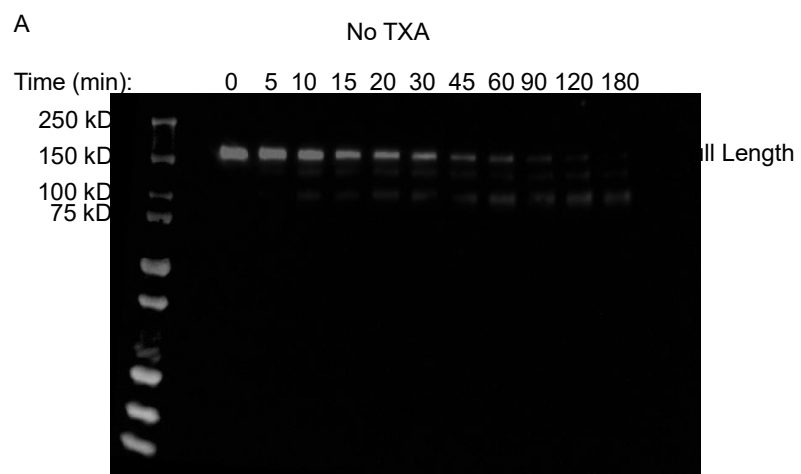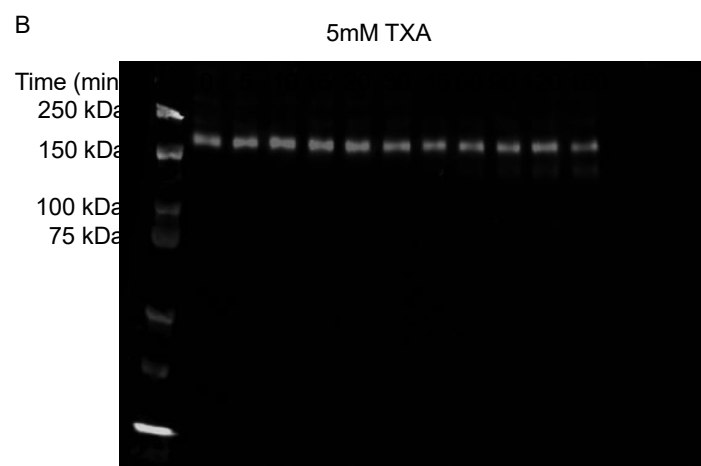

Supplement: Supplementary file 1 — Supplementary Information 1. [file 41598_2024_59672_MOESM1_ESM.pdf]

Figure 1

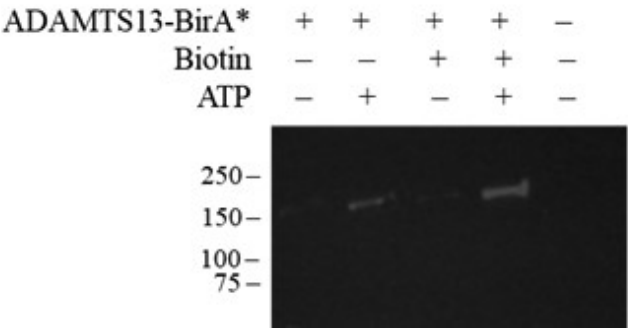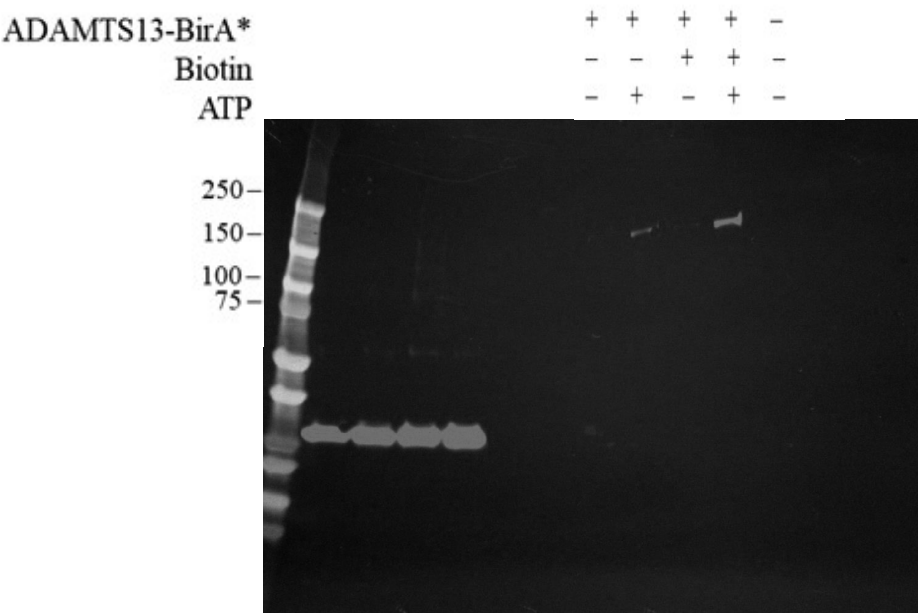

Figure 2B

B

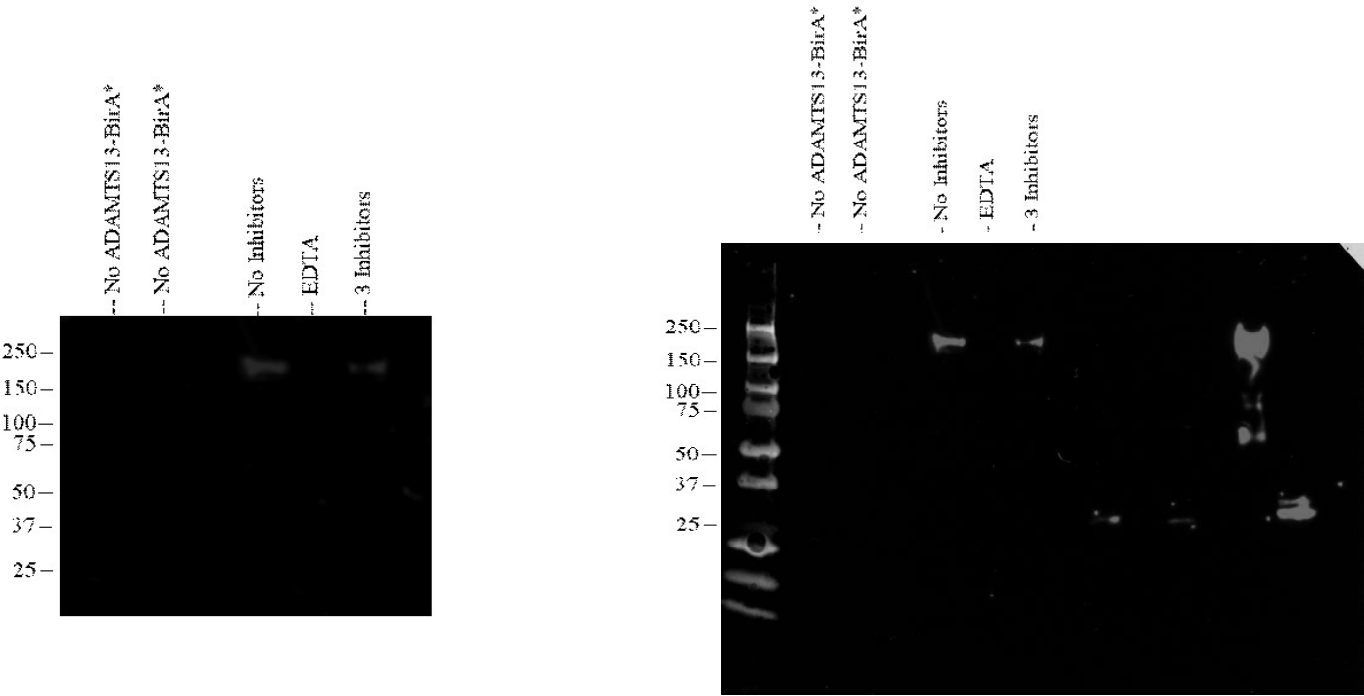

Figure 2C

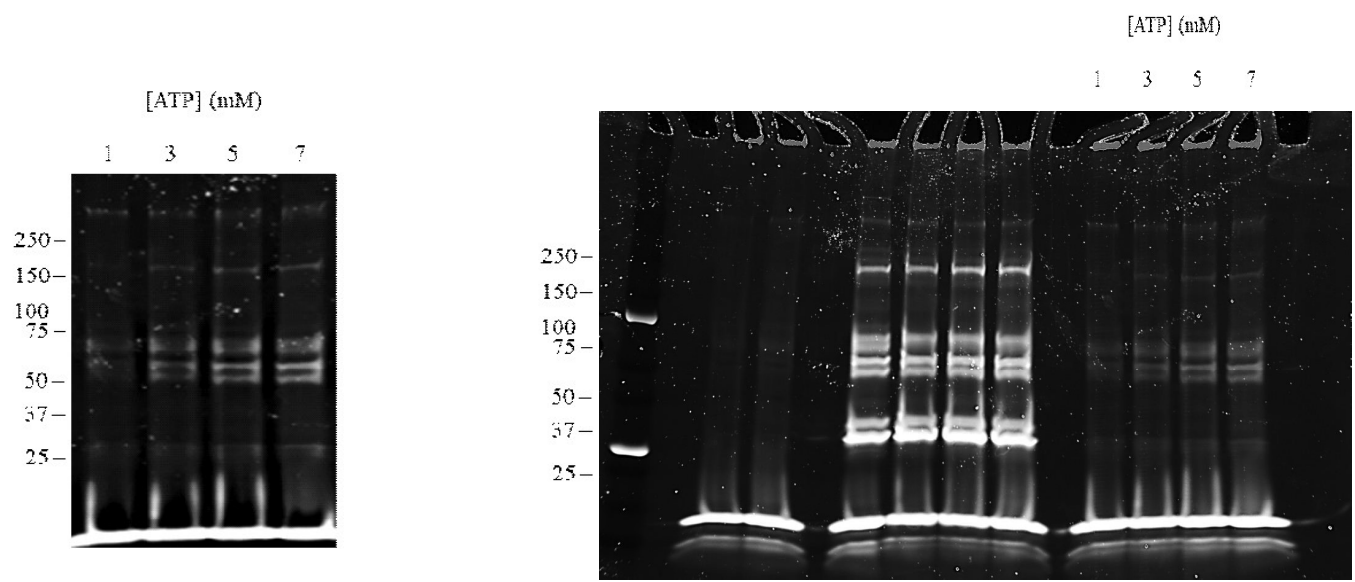

Supplement: Supplementary file 6 — Supplementary Information 6. [file 41598_2024_59672_MOESM6_ESM.pdf]
