# Supplementary material for: Optimization of plasma-based BioID identifies plasminogen as a ligand of ADAMTS13
Source: Sci Rep. 2024 Apr 20;14:9073. doi: 10.1038/s41598-024-59672-6 (PMC11032339; doi:10.1038/s41598-024-59672-6)
Supplement: Supplementary file 3 — Supplementary Information 3. [file 41598_2024_59672_MOESM3_ESM.docx]

**SUMMARY TABLE**

| **What is known**   - ADAMTS13 it not regulated by known mechanisms of protease regulation - BioID is a proximity-dependent biotinylation technique used to identify protein interaction networks in cell |
| --- |
| **What does this paper add?**   - A method for BioID was developed to identify the ADAMTS13 protein interaction network in plasma. - Plasma BioID of ADAMTS13 identified extracellular proteins including VWF and plasminogen. - ADAMTS13 binding to plasminogen is lysine-dependent and TXA protected ADAMTS13 from plasmin degradation |
