# Supplementary material for: Optimization of plasma-based BioID identifies plasminogen as a ligand of ADAMTS13
Source: Sci Rep. 2024 Apr 20;14:9073. doi: 10.1038/s41598-024-59672-6 (PMC11032339; doi:10.1038/s41598-024-59672-6)
Supplement: Supplementary file 4 — Supplementary Information 4. [file 41598_2024_59672_MOESM4_ESM.docx]

**Table 2 -** PCR Conditions in the Extraction of DNA Fragments of Interest

The following is a list of all the primers used in the PCR extraction of the genes of interest and their sequences (5’ to 3’):

**Bold** – primer, non-bolded – overhang, *italicized* – overlap

| **Primer1** | **Primer2** | **Gene** | **Tm (°C)** | | **PCR (°C)** |
| --- | --- | --- | --- | --- | --- |
|  |  |  | P1 | P2 | Extension Time (min:sec) |
| Vector 1 – ADAMTS13-GS-BirA* | | | | | |
| V1P1S  (***AAACCCGCTGATCAGCCTCGA)*** | V1P1AS  (*GCACGAGCCAGCATGGTGGC***TTAAA**  **CGCTAGCCAGCTTGG)** | pcDNA (~5kbps) | 68 | 62.8 | 2:30 |
| V1P2S  (GCTCGGATCT*GCCACC****ATGCT***  ***GGCTCGTGC*ACTG)** | V1P2AS  (*CACAGCGGCCCACACCCA***GGCCTGC**  **CGTGGCTTA)** | Cox + MDTCS (~2kbps) | 65.1 | 64.4 | 1:00 |
| V1P3S  (*TGGGTGTGG****GCCGCTGTG*CGT**  **GGG)** | V1P3AS  (*CCGCTGCCTCCCCCT*GAGCCACCAC  C**GGTTCCTTCCTTTCCCTTCCA)** | TSP+ CUB (~2.25kbps) | 66 | 64 | 1:00 |
| Vector 2 – BirA*-FLAG | | | | | |
| V2P1S  (***AAACCCGCTGATCAGCCTCGA)*** | V2P1AS  (*GCACGAGCCAGCATGGTGGC***TTAAA**  **CGCTAGCCAGCTTGG)** | pcDNA (~5kbps) | 68 | 62.8 | 2:30 |
| V2P2S  (GCTCGGATCT*GCCACC****ATGCTG***  ***GCTCGTGC*ACTG)** | V2P2AS  (*GTTCTTGAA****GGCATTGG*ATTGGAAG**  **TACAG)** | Cox (~0.15kbps) | 65.1 | 64 | 0:30 |
| V2P3S  (*CCAATGCC****TTCAAGAAC*CTGAT**  **CTGGCTGAA)** | V2P3AS  (*ATCGTCTTTGTAGTC*GGAACCACC**GC**  **TTCTTCTCAGGCTGAACTC)** | BioID2 (~0.75kbps) | 64.2 | 62.6 | 0:30 |
