# Supplementary material for: Optimization of plasma-based BioID identifies plasminogen as a ligand of ADAMTS13
Source: Sci Rep. 2024 Apr 20;14:9073. doi: 10.1038/s41598-024-59672-6 (PMC11032339; doi:10.1038/s41598-024-59672-6)
Supplement: Supplementary file 5 — Supplementary Information 5. [file 41598_2024_59672_MOESM5_ESM.docx]

**Table 3 -** PCR Conditions in the Sequencing of the Assembled Junctions

The following is a list of all the primers used in the PCR extraction of the genes of interest and their sequences (5’ to 3’):

**Bold** – primer, non-bolded – overhang, *italicized* – overlap

| **Junction (length – bp)** | **Primer 1** | | **Primer 2** | | **Tm (°C)** | | **PCR (°C)** |
| --- | --- | --- | --- | --- | --- | --- | --- |
|  | Name | Sequenced Region | Name | Sequenced Region | P1 | P2 | Extension (min:sec) |
| **Vector 1 and 4 – ADAMTS13-GS/GSA-BirA*** | | | | | | | |
| **pcDNA/CoxM (~400)** | V1S1F  (TAATACGACTCACTA  TAGGGAGAC) | Vector-Cox-M | V1S1R  (AGGTTGGCTGTGATA  TTTGGA) | M-Cox-Vector | 60.5 | 62.1 | 0:30 |
| **S/TSP (~420)** | V1S2F  (CCTGGAGGAGATCC  GCATCT) | S-TSP | V1S2R  (CAATGTCTTCAGGAG  GCTGCC) | TSP-SC | 65.1 | 65.1 | 0:30 |
| **CUB/BirA* (~390)** | V1S3F  (CAGGTGCTCTACTGG  GAGTCA) | CUB-GS/GSA-BirA*-FLAG-Vector | V1S3R  (TTCTCGAACTCCTTGG  GGTTC) | BirA*-GS/GSA-CUB | 64.9 | 63.8 | 0:30 |
| **BirA*/pcDNA (~360)** | V1S4F  (CCTGAAGAAGTTCA  AGGAGAA) | BirA*-FLAG-Vector | V1S4R  (AGGAAAGGACAGTG  GGAGTGG) | Vector-FLAG-BirA*-GS/GSA-CUB | 59.8 | 65.6 | 0:30 |
| **Vector 8 – BirA*-FLAG** | | | | | | | |
| **pcDNA/BirA* (~370)** | V1S1F  (TAATACGACTCACTA  TAGGGAGAC) | Vector-Cox-BirA*-FLAG-Vector | V1S3R  (TTCTCGAACTC  CTTGGGGTTC) | BirA*-Cox-Vector | 60.5 | 63.8 | 0:30 |
| **BirA*/pcDNA (~360)** | V1S4F  (CCTGAAGAAGTTCA  AGGAGAA) | BirA*-FLAG-Vector | V1S4R  (AGGAAAGGACAGTG  GGAGTGG) | Vector-FLAG-BirA*-Cox-Vector | 59.8 | 65.6 | 0:30 |
